# Spatiotemporal Characterization of the Functional MRI Latency Structure with Respect to Neural Signaling and Brain Hierarchy

**DOI:** 10.1002/advs.202504956

**Published:** 2025-08-27

**Authors:** Hyoungshin Choi, Yeongjun Park, Jong‐eun Lee, Sunghun Kim, Bo‐yong Park, Hyunjin Park

**Affiliations:** ^1^ Department of Electrical and Computer Engineering Sungkyunkwan University Suwon 16419 Republic of Korea; ^2^ Center for Neuroscience Imaging Research Institute for Basic Science Suwon 16419 Republic of Korea; ^3^ Department of Brain and Cognitive Engineering Korea University Seoul 02841 Republic of Korea; ^4^ BK21 Four Institute of Precision Public Health Seoul 02841 Republic of Korea; ^5^ School of Electronic and Electrical Engineering Sungkyunkwan University Suwon 16419 Republic of Korea

**Keywords:** biophysical model, functional connectivity, gradients, intrinsic neural timescale, latency structure

## Abstract

The intrinsic brain activity observed through resting‐state functional magnetic resonance imaging (rs‐fMRI) offers significant information to investigate underlying brain processes. Since traditional latency analysis models are limited to assessing macroscopic functional dynamics, the physical significance of fMRI‐derived latency structures remains unexplored. To fill the gap, the spatiotemporal characteristics of fMRI are investigated using latency structure analysis in 469 neurologically healthy adults. After calculating the lagged cross‐covariance of the time series, principal component analysis is applied to generate latency eigenvectors. These eigenvectors are associated with neural parameters derived from the biophysical model, revealing significant correlations with excitatory and inhibitory synaptic gating, recurrent connection, and excitation/inhibition balance. Association analyses with temporal and spatial features revealed that the latency eigenvectors are significantly associated with intrinsic neural timescale, and each latency eigenvector is paired with major brain axes from functional gradients, including the sensory‐transmodal, visual‐motor, and multiple demand‐task‐negative systems. These findings indicate that the latency model aligns with a seminal model of cortical hierarchy and intrinsic neural signaling. The clinical implications of latency eigenvectors are validated in autism spectrum disorder. This study enhances the understanding of the spatiotemporal characteristics of fMRI signals, providing insights into the physiology underlying the latency structures of brain signals.

## Graphical abstract

Resting‐state fMRI captures intrinsic brain activity, yet the physical significance of latency structures remains unclear. In this study, the spatiotemporal properties of fMRI‐derived latency structures are examined by linking them to biophysical model‐based neural functions, intrinsic neural timescales, and functional gradients. Their clinical relevance in autism spectrum disorder is also explored.

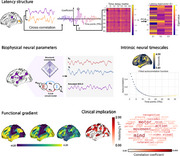

## 1 Introduction

Since the discovery of fluctuations in blood‐oxygen‐level‐dependent (BOLD) signals on functional magnetic resonance imaging (fMRI) reflect intrinsic brain activity, numerous attempts have been made to identify the mechanisms underlying these fluctuations from both spatial and temporal perspectives.^[^ ^1^, ^2^, ^3^, ^4^, ^5^, ^6^, ^7^ ^]^ The most prominent approach involves the examination of functional connectivity to assess the synchronicity of intrinsic brain activity among different brain regions.^[^ ^6^, ^8^, ^9^, ^10^, ^11^ ^]^ This method has defined large‐scale resting‐state networks (RSNs) representing sensory/motor and cognitive control‐related responses,^[^ ^12^, ^13^ ^]^ allowing the elucidation of the spatial patterns associated with spontaneous brain activity in both healthy and pathological brains.^[^ ^14^, ^15^, ^16^, ^17^ ^]^ However, RSNs predominantly offer a spatial perspective, meaning that temporal characteristics are largely unexplored.

The temporal lag or delay approach (sometimes referred to as the lag structure) is one method used to explore temporal dynamics in fMRI.^[^ ^6^, ^18^ ^]^ This approach expresses the latency structure of intrinsic brain activity using a time‐shift property measuring the delay or advance of the time series between brain regions. The analysis revealed that the segregated networks exchange information by propagating intrinsic brain activity on a macroscopic scale.^[^ ^6^ ^]^ Further, the propagation of intrinsic brain activity represents the propagation of changes in neuronal excitability.^[^ ^18^ ^]^ Understanding this process could enhance our knowledge of the physiological characteristics of brain function. Dimensionality reduction techniques have been further applied to project high‐dimensional data onto a low‐dimensional eigenspace to yield multiple eigenvectors.^[^ ^18^ ^]^ This approach led to a succinct representation of the latency structure, which identified spatially overlapping core components in the propagation of intrinsic brain activity.^[^ ^18^ ^]^ Interestingly, they found that the topographies of the core components and RSNs are separable, although they do share some commonalities.^[^ ^6^, ^18^ ^]^ Considering the relationship between the latency structure and RSNs, identifying the topography underlying the latency structure may enhance our understanding of cognitive processes in healthy and diseased brains.^[^ ^19^ ^]^

One of the primary challenges in analyzing latency structures using fMRI is ensuring biological interpretability. Animal studies using optical imaging techniques support the general premise that latency structures reflect biologically grounded neural communications. Studies using voltage‐sensitive dye imaging or calcium imaging in animal models offer millisecond‐level temporal resolution and have been effectively used to analyze fine‐grained latency structures across cortical areas.^[^ ^20^, ^21^ ^]^ A study using rodent fMRI with a repetition time (TR) of 1.5 s showed infraslow cortical propagation (≈0.01–0.1 Hz), consistent with findings from calcium/hemoglobin optical imaging that observed spontaneous infraslow propagations from the motor cortex to the visual cortex in awake mice, reflecting true neural activity.^[^ ^22^, ^23^ ^]^ Nevertheless, the inherently low temporal resolution of fMRI poses significant difficulties in establishing direct correspondences between macroscale temporal dynamics and neural‐level processes. Biophysical modeling offers a computational framework for simulating neural processes based on macroscale connectome data, integrating physiological parameters that regulate brain dynamics.^[^ ^24^, ^25^, ^26^ ^]^ This approach mitigates the inherent limitations of fMRI in capturing fine‐grained temporal fluctuations. In particular, the neural mass model provides a mathematical framework for characterizing the collective dynamics of neuronal populations.^[^ ^25^, ^26^ ^]^ Rather than modeling individual neurons, this model estimates the average activity of neuronal ensembles by incorporating interactions between excitatory and inhibitory neurons. It facilitates the quantification of key neural functions, such as excitatory/inhibitory (E/I) balance, synaptic interactions, and signal propagation.^[^ ^24^, ^27^ ^]^ By integrating biophysical models with fMRI‐derived latency structures, we can enhance their biological interpretability, enabling a more mechanistic understanding of how macroscale temporal dynamics reflect underlying neural mechanisms.

Another representative method for exploring temporal dynamics in fMRI is the intrinsic neural timescale (INT), which describes the duration over which neural activity in a specific brain region correlates with itself.^[^ ^28^ ^]^ This property is linked to functional specialization and neurophysiological processes.^[^ ^28^ ^]^ Brain regions with longer timescales excel at integrating higher‐order information, particularly in tasks involving short‐term memory or decision‐making processes.^[^ ^29^, ^30^ ^]^ These brain regions further exhibit stronger synaptic connections, characterized by robust recurrent connections, which contribute to the slow accumulation of information for decision‐making processes.^[^ ^28^, ^29^, ^30^ ^]^ As such, the spatial patterns of INT appear hierarchically across cortical areas along the cognitive and sensory axes.^[^ ^31^, ^32^, ^33^ ^]^ Simply put, INT represents the decay of autocorrelation with increasing time latency, and thus is a complementary measure of the latency structure. As such, exploring the association between the latency structure and INT can help identify the underlying properties of intrinsic brain activity.

The gradient technique is a well‐suited approach for analyzing RSNs, as it describes the hierarchical properties of brain functions.^[^ ^34^ ^]^ This approach applies a dimensionality reduction technique to reduce the dimensionality of the connectivity matrix to multiple eigenvectors.^[^ ^34^ ^]^ The estimated eigenvectors in a low‐dimensional space, commonly referred to as gradients, offer valuable insights into the spatial organization of large‐scale brain networks, effectively illustrating a spectrum of brain hierarchies that span unimodal to heteromodal association brain systems.^[^ ^34^ ^]^ Typically, the first gradient spans from the primary sensory to association cortices, while the second extends from the visual to the somatomotor system, and the third extends from the multiple demand network to task‐negative systems.^[^ ^34^, ^35^, ^36^, ^37^ ^]^ The utility of functional gradients has been demonstrated in previous studies through the assessment of the cognitive states associated with atypical neurodevelopment^[^ ^38^, ^39^, ^40^ ^]^ and healthy aging.^[^ ^36^, ^41^ ^]^

In this study, we estimated the latency structure using resting‐state fMRI (rs‐fMRI) data and explored its biological underpinnings based on the neural functions derived from the biophysical models. Then, we examined the relationship between the latency structure, INT (i.e., temporal aspect), and functional gradients (i.e., spatial aspect) to identify a subtle association that may be obscured if only the latency structure was considered, by leveraging the complementary information of the INT and functional gradients. Finally, we explored the clinical implications of latency eigenvectors in a neuropsychiatric condition of autism spectrum disorder (ASD).

## 2 Results

### 2.1 Latency Structure of the Functional Time Series

**Figure 1:**
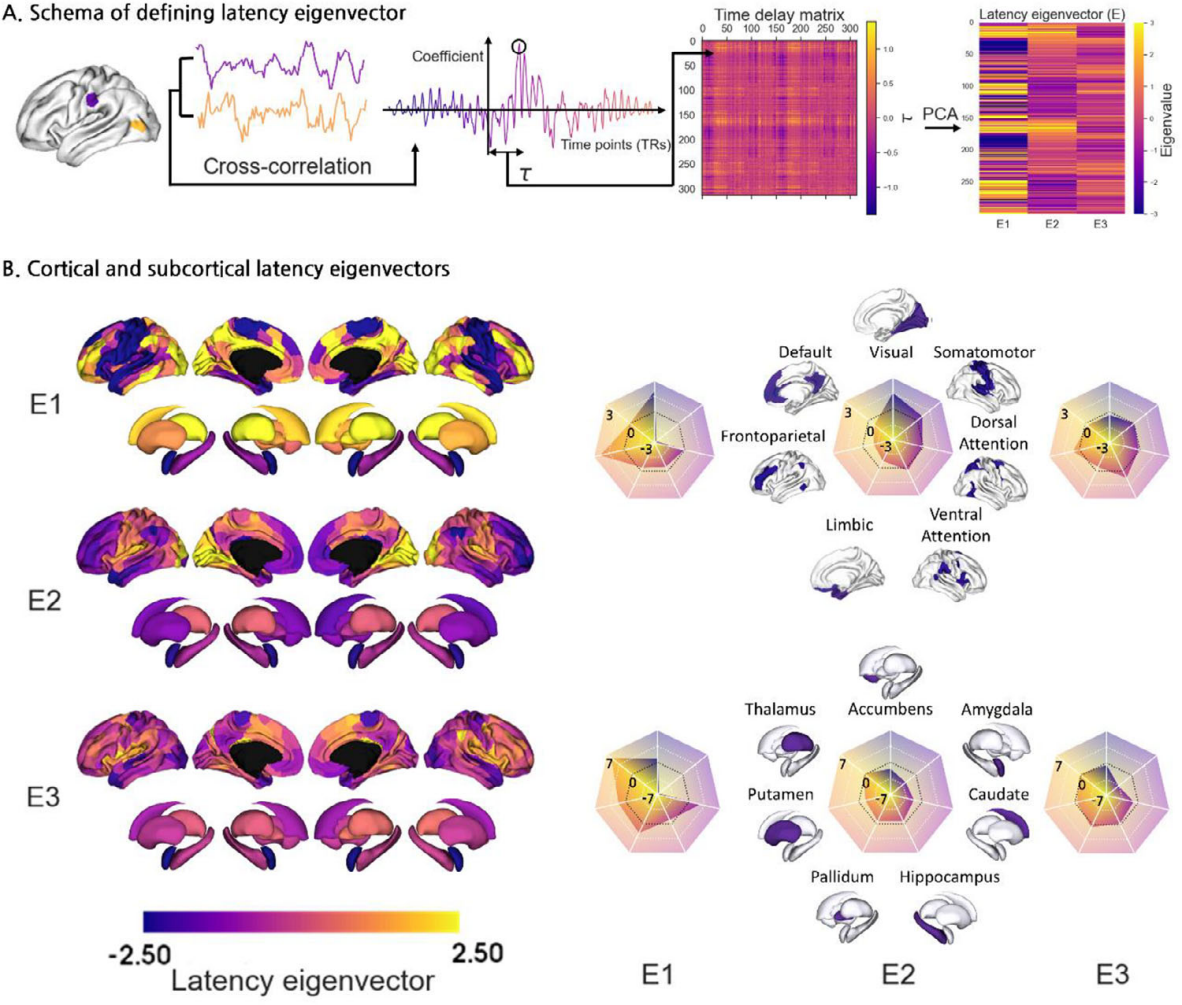
Computation of latency eigenvectors. A) Schematic illustration of computing latency between two different brain regions (left). The time delay (τ) is derived by maximizing the cross‐correlation coefficient between the time series of two different regions, and it is entered into the time delay matrix (middle). Latency eigenvectors are generated using dimensionality reduction techniques, and three dominant eigenvectors (E1, E2, E3) are selected (right). B) Visualization of the first three latency eigenvectors on the cortical surfaces and subcortical structures (left). The values of the eigenvectors are stratified according to seven intrinsic functional communities and each subcortical area (right). *Abbreviation*: PCA, principal component analysis.

We studied rs‐fMRI data of 469 genetically unrelated participants from the Human Connectome Project (HCP) database.^[^ ^42^ ^]^ The time delay matrix was calculated based on the lagged cross‐covariance of the time series between different brain regions^[^ ^6^, ^18^ ^]^ defined using the Schaefer atlas with 300 parcels^[^ ^43^ ^]^ and 14 subcortical areas from the Desikan–Killiany atlas.^[^ ^44^ ^]^ Then, the latency eigenvectors were generated by applying principal component analysis (PCA) to the time delay matrix (**Figure** **1**A). The first three eigenvectors, which explained ≈88% of the original data, were considered (Figure S1, Supporting Information). In the first latency eigenvector, the negative‐to‐positive axis was constructed by the somatomotor cortex and amygdala (negative), and the visual and frontoparietal cortices and thalamus (positive). The second latency eigenvector revealed an axis from the transmodal (i.e., frontoparietal and default mode networks) cortex and striatum to the sensory/motor regions. The third latency eigenvector exhibited an axis from the limbic cortex and amygdala to the control systems (Figure 1B).

### 2.2 Neural Correlates of the Latency Structure

**Figure 2:**
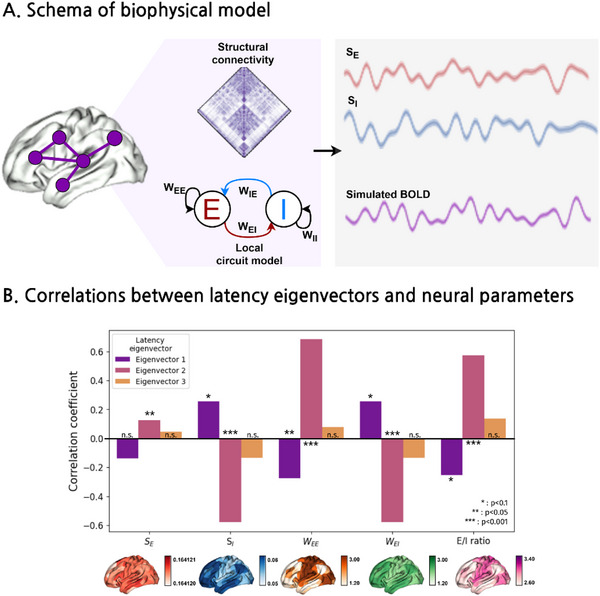
Associations of latency eigenvectors with biophysical model‐derived neural functions. A) A schema of the parametric feedback inhibition control (pFIC) model. From the macroscale structural connectivity matrix, recurrent connections and synaptic gating parameters, as well as simulated BOLD signal, were estimated. B) Correlations between latency eigenvectors and neural parameters are shown. *Abbreviations*: BOLD, Blood‐oxygenation‐level‐dependent; ***S***_***E***_, excitatory synaptic gating; ***S***_***I***_, inhibitory synaptic gating; ***W***_***EE***_, excitatory‐to‐excitatory recurrent connection; ***W***_***EI***_, excitatory‐to‐inhibitory connection; E/I ratio, excitatory/inhibitory ratio; n.s., not significant.

The parametric feedback inhibitory control (pFIC) model was employed to characterize neural dynamics, generating excitatory (*S_E_*) and inhibitory synaptic gating (*S_I_*) variables, along with excitatory‐to‐excitatory (*W_EE_*) and excitatory‐to‐inhibitory connection strengths (*W_EI_*), as well as the E/I balance^[^ ^24^ ^]^ (**Figure** **2**A). We associated latency eigenvectors with neural parameters using 1000 spin permutation tests^[^ ^45^ ^]^ and a false discovery rate (FDR) to account for spatial autocorrelation and correct for multiple comparisons^[^ ^46^ ^]^ (Figure 2B). The second latency eigenvector, which exhibited a sensory‐transmodal hierarchy, was positively correlated with *S_E_* (r = 0.13, p_spin‐FDR_ = 0.03), *W_EE_* (r = 0.68, p_spin‐FDR_ < 0.001), and E/I ratio (r = 0.57, p_spin‐FDR_ < 0.001), and negatively correlated with *S_I_* (r = −0.58, p_spin‐FDR_ < 0.001) and *W_EI_* (r = −0.58, p_spin‐FDR_ < 0.001). In contrast, the first latency eigenvector, which mapped onto the somatomotor‐visual/frontoparietal axis, exhibited opposite patterns, showing moderate positive correlations with *S_I_* (r = 0.25, p_spin‐FDR_ = 0.07) and *W_EI_* (r = 0.25, p_spin‐FDR_ = 0.07), while other parameters exhibited negative correlations (*W_EE_*: r = −0.27, p_spin‐FDR_ < 0.001, and E/I ratio: r = −0.25, p_spin‐FDR_ = 0.07). These findings indicate that latency eigenvectors are differentially associated with excitatory and inhibitory neural functions.

Additionally, we assessed the reliability of the estimated latency eigenvectors by comparing their spatial patterns between actual and simulated data with faster TRs of 0.36, 0.18, and 0.09 s. Significant correlations between corresponding eigenvectors across different TRs were observed in latency eigenvectors 1 and 2 (mean ± standard deviation [SD] r = 0.36 ± 0.02, p_spin‐FDR_ = 0.01 for latency eigenvector 1; r = 0.44 ± 0.05, p_spin‐FDR_ < 0.001 for latency eigenvector 2), while the third latency eigenvector showed relatively low correspondence (Figure S2, Supporting Information). These results reinforce the reliability of estimating latency eigenvectors from fMRI data.

### 2.3 Associations Between the Latency Structure and Intrinsic Neural Timescale

**Figure 3:**
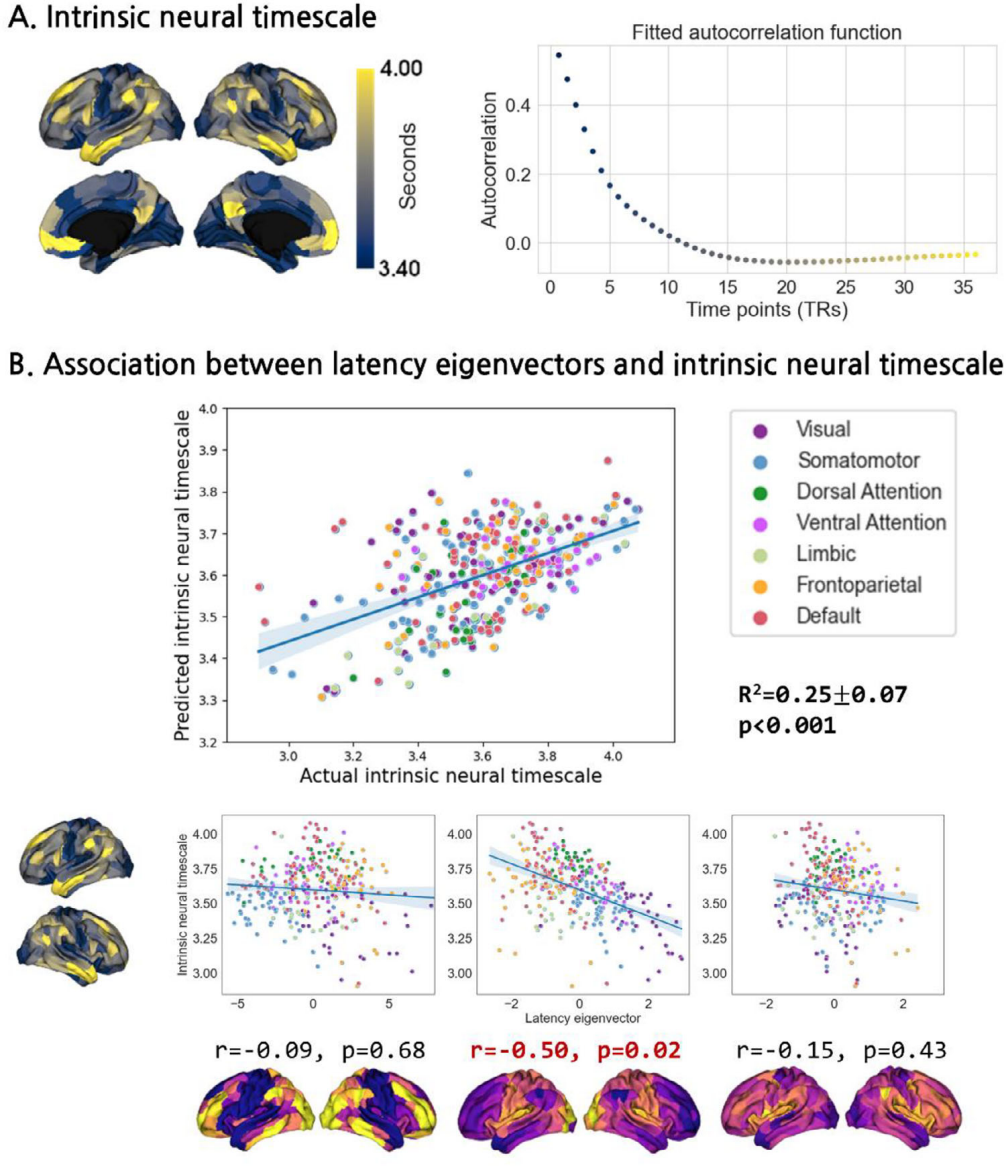
Associations between latency eigenvectors and the intrinsic neural timescale. A) The spatial pattern of the intrinsic neural timescale is shown on the brain surfaces (left), and the scatter plot on the right indicates the fitted autocorrelation function across the repetition time (TR). B) The scatter plot on the first row represents the linear regression between the three latency eigenvectors and the intrinsic neural timescale, where the shaded regions indicate the confidence interval across a five‐fold cross‐validation. The plots below represent the correlations between each latency eigenvector and intrinsic neural timescale. Red bold font indicates a significant association.

To assess temporal characteristics of the latency eigenvectors, we associated them with INT, which was estimated by fitting a nonlinear exponential decay function to the autocorrelation function. INT showed longer timescales in higher‐order transmodal regions, whereas unimodal association areas showed shorter timescales (**Figure** **3**A). Linear regression with a five‐fold cross‐validation revealed significant associations between the three latency eigenvectors and INT (mean ± SD = 0.25 ± 0.07, p_spin‐FDR_ < 0.001; Figure 3B). When we associated each latency eigenvector and INT, only the second latency eigenvector showed a significant correlation (r = −0.50, p_spin‐FDR_ = 0.02; Figure 3B). This finding indicates that the latency eigenvector showing a clear sensory‐transmodal cortical hierarchy may be particularly related to intrinsic neural signaling, where information in the sensory/motor regions propagates more rapidly than in the transmodal regions.

### 2.4 Associations with Functional Gradients

**Figure 4:**
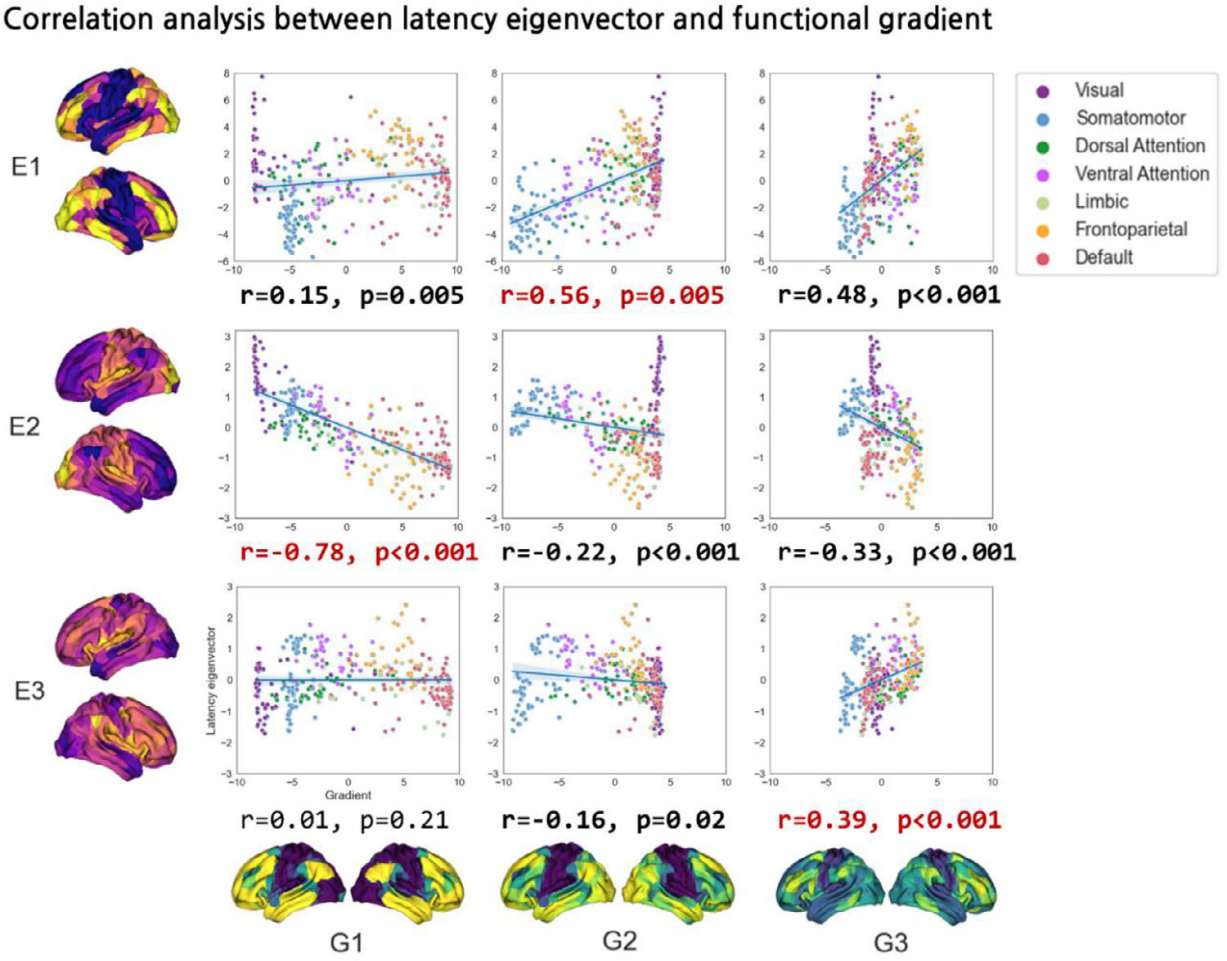
Associations between latency eigenvectors and functional gradients. The correlations between latency eigenvectors (E1–3) and functional gradients (G1–3) are shown. Red bold font indicates the highest associations, while black bold font represents significant associations.

To characterize the spatial topology of the latency eigenvectors, association analysis with functional gradients was performed (**Figure** **4**). The first latency eigenvector showed the strongest association with the second functional gradient of the motor‐visual axis (r = 0.56, p_spin‐FDR_ = 0.005), the second latency eigenvector with the first functional gradient of the sensory‐transmodal axis (r = −0.78, p_spin‐FDR_ < 0.001), and the third latency eigenvector with the third functional gradient of the negative‐multiple demand network axis (r = 0.39, p_spin‐FDR_ < 0.001). Overall, our findings revealed strong links between latency eigenvectors and functional gradients, indicating that latency eigenvectors may follow a seminal model of the cortical hierarchy.

### 2.5 Clinical Implications in Autism Spectrum Disorders

**Figure 5:**
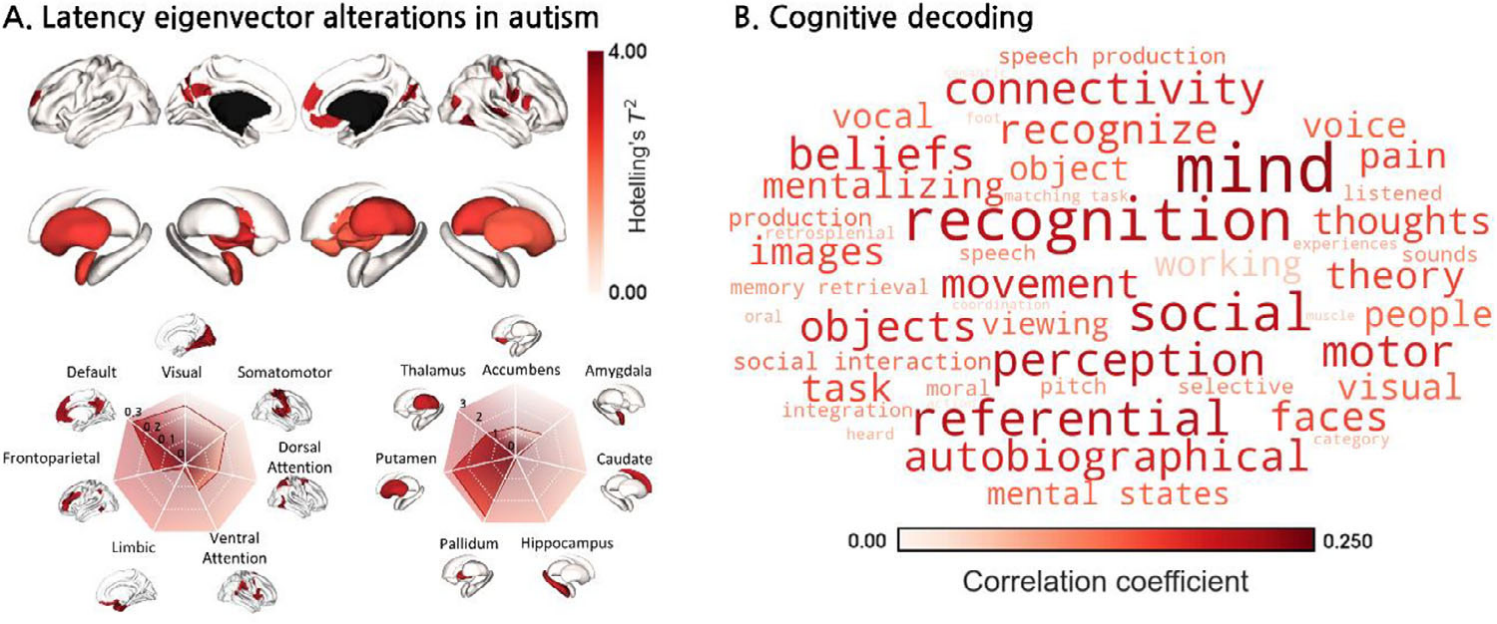
Between‐group differences in the cortical and subcortical latency eigenvectors and cognitive decoding analysis. A) Between‐group differences in the latency eigenvectors are visualized on the cortical and subcortical surfaces. Effects are stratified according to seven intrinsic functional communities and each subcortical structure. B) Results of the cognitive decoding analysis using NeuroSynth are presented as a word cloud.

Given the importance of the latency structure in cognitive processing, we examined the clinical implications of latency eigenvectors using rs‐fMRI data from 208 individuals with ASD and 241 typically developing (TD) controls obtained from the Autism Brain Imaging Data Exchange‐I (ABIDE‐I) database.^[^ ^47^ ^]^ When we compared the latency eigenvectors between the groups, significant differences (p_spin‐FDR_ < 0.05) were identified in the precuneus, medial prefrontal cortex, and somatomotor regions, involved in the default mode and visual/somatomotor networks, as well as in the thalamus, putamen, pallidum, accumbens, and amygdala (**Figure** **5**A). To determine the cognitive associations underlying the differences in cortical and subcortical latency eigenvectors between the groups, we performed meta‐analysis cognitive decoding.^[^ ^48^, ^49^ ^]^ We found correlations with cognition‐ and sensory‐related terms, such as “mind,” “recognition,” “perception,” “autobiographical,” “social,” “motor,” and “task” (Figure 5B).

### 2.6 Sensitivity Analysis Using Different Parcellations

To assess the robustness of the findings across different parcellation schemes, we repeated the generation of latency eigenvectors and associated them with the INT and functional gradients using the Schaefer atlas with 200 and 400 parcels and the Glasser atlas with 360 parcels. When we compared the spatial maps of the latency eigenvectors on the vertices, we observed largely consistent patterns (r = 0.89 ± 0.03, p_spin‐FDR_ < 0.001 for Schaefer 200; r = 0.91 ± 0.02, p_spin‐FDR_ = 0.003 for Schaefer 400; r = 0.78 ± 0.08, p_spin‐FDR_ < 0.001 for Glasser 360). Associations between INT and functional gradients also showed comparable results (Figures S3–S5, Supporting Information).

### 2.7 Sensitivity Analysis Using Different Latency Thresholds

We repeated the generation of latency eigenvectors and the associations with INT and functional gradients by applying 3 and 1.5 s thresholds to the time delay matrix. When we compared the spatial maps of the latency eigenvectors, largely consistent patterns were observed (r = 0.97 ± 0.01, p_spin‐FDR_ = 0.003 for 3 s threshold; r = 0.77 ± 0.21, p_spin‐FDR_ = 0.02 for 1.5 s threshold; Figures S6A and S7A, Supporting Information). Associations of latency eigenvectors with INT and functional gradients also showed comparable results (Figures S6B,C and S7B,C, Supporting Information). However, the third latency eigenvector showed noticeable variation when a 1.5 s threshold was applied, indicating that it may encode slower distributed propagation dynamics, which could be more sensitive to longer latency structures.

### 2.8 Sensitivity Analysis Using a Different Dimensionality Reduction Technique

We applied diffusion map embedding to the time delay matrix,^[^ ^50^ ^]^ instead of PCA, and repeated the analyses. The spatial maps of the latency eigenvectors were largely consistent (r = 0.99 ± 0.01, p_spin‐FDR_ < 0.001; Figure S8A, Supporting Information), and the associations with INT and functional gradients were also comparable (Figure S8B,C, Supporting Information), indicating the robustness of our features with respect to dimensionality reduction techniques.

## 3 Discussion

Understanding the spatiotemporal properties of intrinsic brain activities can provide insights into the underlying neurophysiology. Considering the importance of the propagation of intrinsic brain activity, we systematically investigated the spatiotemporal properties of latency structures. Leveraging the lagged cross‐covariance approach^[^ ^6^, ^18^ ^]^ and dimensionality reduction techniques, we generated three latency eigenvectors in both the cortical and subcortical regions. The estimated latency eigenvectors were significantly related to neural parameters derived from the biophysical model. Association analysis with the INT confirmed that the latency eigenvector showing a sensory‐transmodal cortical hierarchy is related to intrinsic neural signaling. Moreover, the latency eigenvectors were highly associated with functional gradients, indicating that the latency model aligned with the seminal model of the cortical hierarchy. Furthermore, a comparison between individuals with ASD and TD revealed that latency eigenvectors could be used as a compact, biologically informed marker to describe atypical neural dynamics in psychiatric conditions.

Latency eigenvectors represent major components of the latency structure, effectively retaining substantial information from the original time delay matrix, but with a reduced number of features.^[^ ^18^ ^]^ Negative and positive values of the latency eigenvectors may represent the distribution of intrinsic brain activity across brain regions.^[^ ^18^ ^]^ The spatial pattern of the first latency eigenvector discriminated the somatomotor cortex and amygdala, integrating motor planning and emotional responses from the visual and frontoparietal networks and the thalamus, which are related to adaptive processes and decision‐making,^[^ ^51^, ^52^, ^53^ ^]^ indicating a bottom‐up process.^[^ ^18^ ^]^ The second latency eigenvector propagated between higher‐order transmodal regions and low‐level sensory/motor areas, indicating a typical top‐down process.^[^ ^54^, ^55^, ^56^, ^57^ ^]^ The third latency eigenvector potentially indicated the involvement of a dynamic interplay between the sensory, emotional, and cognitive processes in the brain, facilitating adaptive behavior and effective cognitive functioning.^[^ ^58^, ^59^, ^60^ ^]^

The relationship between biophysical model‐derived neural parameters and latency eigenvectors provides insights into the neural basis of temporal dynamics. Our findings demonstrated that the latency eigenvectors were significantly correlated with excitatory and inhibitory mechanisms, with the sensory‐transmodal latency eigenvector showing particularly strong associations with neural parameters. These results suggest that intrinsic neural activity may be closely linked to temporal dynamics at the macroscale. Given that the E/I ratio plays a crucial role in regulating signal propagation and network stability,^[^ ^61^, ^62^ ^]^ it is noteworthy that latency structures may be shaped by fundamental biophysical properties of the brain. This reinforces the potential of latency eigenvectors as a biologically meaningful metric for capturing large‐scale temporal dynamics within brain systems. Together, our results offer compelling neurobiological evidence supporting the interpretability of latency eigenvectors in understanding the temporal dynamics of brain networks.

In addition to the neural functions, we compared the latency eigenvectors with the INT to impart additional physical meaning to the latency structure. INT describes the duration over which neural activity in a specific brain region correlates with itself, which is linked to functional specialization and neurophysiological processes for integrating and segregating information.^[^ ^28^ ^]^ Notably, the spatial patterns of INT appear hierarchically across cortical areas, with longer timescales in heteromodal association regions and shorter timescales in sensory areas.^[^ ^28^, ^31^, ^32^, ^33^, ^63^, ^64^ ^]^ This represents the temporal hierarchy of information processing in cortical areas, where the frontoparietal and default mode regions require more time to effectively retain and integrate complex information, while somatomotor and visual areas process information relatively fast.

We also assessed the spatial properties of the latency eigenvectors based on functional gradients. In recent years, manifold learning techniques have become more prevalent for mapping macroscale functional connectome organization,^[^ ^38^, ^65^, ^66^, ^67^, ^68^ ^]^ and it is anticipated that these methods will be able to effectively describe the propagation of intrinsic brain activity. In our analysis, each latency eigenvector was paired with well‐defined functional gradients, suggesting that latency eigenvectors can explain the functional hierarchy of the brain. The complete alignment with a seminal model of neural organization and laminar differentiation containing four cortical hierarchical levels^[^ ^69^ ^]^ indicates that the latency structure of spontaneous brain activity has commonalities in spatial patterns with the hierarchical organization of brain systems.

In particular, the second latency eigenvector showed a significant correlation with INT, indicating that it represents the pattern of temporal hierarchy. Furthermore, the second latency eigenvector was significantly associated with the first functional gradient, which represents the spatial brain hierarchy along the unimodal‐to‐transmodal axis. Together, the propagation dynamics represented by the second latency eigenvector have the potential for explaining spatiotemporal hierarchies of information processing. The hierarchical organization of the brain is a primary principle that constrains cognitive processes.^[^ ^34^, ^70^ ^]^ Indeed, this principal axis was highly associated with social cognition, communication, and reward circuits in healthy and diseased populations of autism, epilepsy, and obesity.^[^ ^37^, ^65^, ^71^, ^72^, ^73^, ^74^, ^75^, ^76^, ^77^, ^78^, ^79^, ^80^ ^]^ We thus believe that the second latency eigenvector may serve a critical role in multiple cognitive functions.

The clinical implications of the latency eigenvectors were explored through an investigation of brain alterations associated with ASD.^[^ ^81^, ^82^ ^]^ Our findings confirm the potential clinical utility of the propagation of intrinsic brain activity for psychiatric conditions. In future studies, more in‐depth investigations associating latency eigenvectors with E/I imbalance should be performed to better understand the multiscale perspective of autism pathophysiology. Moreover, disease classification and symptom prediction need to be systematically explored in multiple psychiatric and neurological conditions to demonstrate the potential of latency eigenvectors as biomarkers.

Our results reframe the latency structure as a biologically meaningful reflection of how information is dynamically routed and integrated across the cortex. Building upon the previous studies that primarily described latency structures in rs‐fMRI in terms of signal propagation,^[^ ^6^, ^18^ ^]^ our work tried to probe the potential underlying biological and computational relationships that govern these latency patterns. First, we demonstrated that the latency eigenvectors aligned with two well‐established macroscale features of brain organization, both in spatial and temporal perspectives: functional connectivity gradients (spatial) and INT (temporal). This triadic correspondence allows us to interpret latency eigenvectors as features reflecting the hierarchical spatiotemporal organization of the brain. However, when the latency threshold was shortened, the association between the third eigenvector (i.e., limbic‐sensory axis) and the third gradient (task negative‐multiple demand network axis) was reduced. The result suggests that multiple demand networks are more evident in longer, slower temporal dynamics settings. Future research should quantitatively examine the differences in eigenvector axes across latency thresholds to assess the effects of fast and slow temporal dynamics and to confirm the cognitive roles associated with them. Second, rather than treating the latency eigenvectors as data‐driven features obtained through dimensionality reduction, we interpreted them as large‐scale modes of information flow that may be shaped by spatial variations in physiological properties by associating with neuronal functions (i.e., synaptic gating and excitation/inhibition ratio). Finally, the latency eigenvectors are interpretable and reproducible across datasets. We demonstrated this with preliminary results using the ABIDE dataset, suggesting that latency eigenvectors can serve as a compact, biologically informed marker for an atypical neurodevelopmental condition. In sum, our contributions lie in providing plausible neural mechanisms underlying the latency eigenvectors.

Acknowledging the limitations of this study is crucial. First, the temporal resolution of fMRI is insufficient to fully elucidate the propagation patterns of intrinsic brain signals. Although we validated the reliability of the latency eigenvectors using simulated BOLD signals with faster TRs, the impact of this issue should be mitigated through the application of better MRI sequences (e.g., faster TR). The computation of fMRI latency requires consideration of sampling errors.^[^ ^83^ ^]^ For example, it has been shown that head motion correction can affect the magnitude of correlation, leading to altered latency structure. Although the HCP data provided fMRI with fast TR (0.72 s), which might mitigate the effects of sampling error,^[^ ^84^ ^]^ it should be noted that alternative methods should be developed to reduce the sampling error while calculating the latency structure in the fMRI data. Moreover, the effects of slice timing correction on latency structure need to be validated in future work. Third, our results linking latency eigenvectors to the parameters derived from the biophysical model do not establish a causal relationship between the macro and microscale observations. Due to the limited temporal resolution of fMRI, demonstrating causal relationships between regions requires complementary methods, such as electrophysiology or optical imaging in animal models. Several prior studies using invasive recordings in animals have shown that microscale spatiotemporal propagation patterns can produce meso and macroscale signal delays measurable with hemodynamic response‐based methods.^[^ ^20^, ^21^ ^]^ Incorporating such multimodal evidence in future work may help strengthen the causal interpretation of latency‐based analyses. Fourth, study participants in the current study are mostly young adults. Future studies should consider including participants with a wider age range, encompassing older individuals, possibly using a larger dataset. Finally, potential site heterogeneity may remain when comparing TD and ASD groups, even after controlling for site effects in the eigenvectors. This is one of the significant challenges in multisite studies,^[^ ^85^, ^86^ ^]^ and thus, the results require cautious interpretation. In future work, we plan to adopt more advanced deep learning‐based methods^[^ ^87^ ^]^ to control for site effects.

In sum, we explored the latency structure of spontaneous brain activity and interpreted the identified latency eigenvectors in relation to neural functions (i.e., biophysical model), as well as the temporal (i.e., INT) and spatial (i.e., functional gradients) aspects of the brain. Overall, our analysis integrating the latency structure, neural function, INT, and functional gradients effectively established a comprehensive framework that captures the spatiotemporal characteristics of fMRI signals. Our findings offer valuable insights into latency structures, and once the analysis has matured, we anticipate that it could facilitate the identification of biomarkers for various neurological and psychiatric conditions.

## 4 Experimental Section

### Study Participants—HCP

Multimodal MRI data were obtained from the S1200 release of the HCP neuroimaging database,^[^ ^42^ ^]^ an extensive research initiative funded by the National Institutes of Health (NIH). Among the 1206 participants, those who were genetically related (i.e., twins) or had a family history of mental illness, neurological disorders, other metabolic syndromes, or drug abuse were excluded. After the application of these criteria, 469 participants were selected for this study (mean ± SD age = 28.16 ± 3.93 years; 48% female; **Table** **1**). The HCP dataset was anonymized, and the data collection procedure was performed as part of the HCP under the guidelines of the Washington University Institutional Review Board.

**Table 1:** Demographic information of study participants.

Categories	HCP	CALTECH		CMU		NYU		PITT	
		ASD	TD	ASD	TD	ASD	TD	ASD	TD
Subject number	469	13	14	11	13	73	98	23	21
Sex (female:male)	223:246	3:10	2:12	2:9	3:10	9:64	24:74	4:19	3:18
Age	28.2 ± 3.9 (22–37)	26.3 ± 8.2 (17.5–45.1)	28.8 ± 9.9 (18.7–44.2)	26.5 ± 6.2 (19–39)	26.9 ± 5.7 (20–40)	14.9 ± 7.1 (7.1–39.1)	15.9 ± 6.4 (6.5–31.8)	19.2 ± 7.4 (11.4–35.2)	19.4 ± 6.4 (12.2–33.2)

Categories		TRINITY		UM_2		USM		YALE	
		ASD	TD	ASD	TD	ASD	TD	ASD	TD
Subject number	—	22	22	10	15	37	37	19	21
Sex (female:male)	—	0:22	0:22	1:9	1:14	0:37	0:37	6:13	7:14
Age	—	17.7 ± 3.4 (13.6–25.9)	17.7 ± 3.7 (12–25.7)	15.1 ± 1.6 (13.1–17.4)	17.6 ± 4.4 (13.6–28.8)	22.5 ± 7.2 (11.4–42.3)	22.4 ± 7.4 (10–39.4)	13.3 ± 3.2 (7–17.8)	13.1 ± 2.8 (8.4–17.8)

Abbreviations: ASD, autism spectrum disorder; TD, typical development; HCP, Human Connectome Project; CALTECH, California Institute of Technology; CMU, Carnegie Mellon University; NYU, New York University Langone Medical Center; PITT, University of Pittsburg School of Medicine; TRINITY, Trinity Centre for Health Sciences; UM_2, University of Michigan: Sample 2; USM, University of Utah School of Medicine; YALE, Yale Child Study Center.

### Study Participants—ABIDE‐I

The multicenter MRI data obtained from the ABIDE‐I database were further analyzed.^[^ ^47^ ^]^ Inclusion criteria included i) participants with moderate‐to‐small head motion (framewise displacement < 0.3 mm), ii) sites that included >10 individuals in the ASD and TD controls, and iii) sites with a TR of 2000 ms or less. Finally, 449 participants from eight sites were selected for this study (mean ± SD age = 18.40 ± 7.53 years; 14% female; Table 1). The diagnosis of autism was confirmed using the Diagnostic and Statistical Manual of Mental Disorders, Fifth Edition (DSM‐5) criteria. There were no significant differences in age (t = 0.52, *p* = 0.60) or sex (χ^2^ = 7.24, *p* = 0.007) between the ASD and TD groups. The ABIDE‐I data collection procedure was performed in accordance with the Health Insurance Portability and Accountability Act guidelines and the 1000 Functional Connectomes Project or Instrument Neutral Distributed Interface protocols.

### MRI Acquisition

All sites provided T1‐weighted MRI and rs‐fMRI data scanned using 3‐T Siemens (HCP, CALTECH, CMU, NYU, PITT, USM, and YALE), Philips (TRINITY), and GE (UM_2) scanners. The HCP database also provided T2‐weighted MRI and diffusion MRI acquired using a 3‐T Siemens scanner. Detailed MRI acquisition parameters are described in Table S1 (Supporting Information).

### Data Preprocessing—HCP

Minimally preprocessed HCP data using FSL, FreeSurfer, and Workbench tools were used.^[^ ^88^, ^89^, ^90^ ^]^ In brief, T1‐ and T2‐weighted data were corrected for gradient nonlinearity and b0 distortions, followed by co‐registration using a rigid‐body transformation. The bias field was corrected using the inverse intensities from T1‐ and T2‐weighting. The processed data were nonlinearly registered in the Montreal Neurological Institute (MNI152) standard space. White and pial surfaces were generated following the boundaries between different tissues.^[^ ^91^, ^92^, ^93^ ^]^ These surfaces were then averaged to generate a mid‐thickness surface, which was subsequently used to generate an inflated surface. The generated spherical surface was registered to the Conte69 template using MSMAll.^[^ ^94^ ^]^

The rs‐fMRI data were corrected for gradient distortions and head motion. The data was registered onto the T1‐weighted structural data and then onto the MNI152 standard space. Magnetic field bias correction, non‐brain tissue removal, and intensity normalization were performed. Noise components attributed to head movement, white matter, cardiac pulsation, and arterial and large vein‐related contributions were removed using the FMRIB ICA‐based Xnoiseifier (FIX).^[^ ^95^ ^]^ Preprocessed rs‐fMRI data were mapped to a standard grey ordinate space using a cortical ribbon‐constrained volume‐to‐surface mapping algorithm.

MRtrix3 was utilized to preprocess the diffusion MRI data to correct for susceptibility distortions, head motion, and eddy currents.^[^ ^96^ ^]^ Structural connectomes were constructed through probabilistic tractography. Tissue types, including cortical and subcortical gray matter, white matter, and cerebrospinal fluid, were defined from T1‐weighted images, and anatomically constrained tractography was performed.^[^ ^97^ ^]^ After aligning the T1‐weighted data to the native diffusion MRI space via boundary‐based registration, the transformation was applied to different tissue types. Multi‐shell and multi‐tissue response functions were estimated,^[^ ^98^ ^]^ followed by constrained spherical deconvolution and intensity normalization.^[^ ^99^ ^]^ A tractogram containing 40 million streamlines was generated, with a maximum tract length of 250 and a fractional anisotropy threshold of 0.06. Spherical‐deconvolution informed filtering of tractograms (SIFT2) was applied to produce whole‐brain streamlines weighted by cross‐section multipliers.^[^ ^100^ ^]^ The resulting streamlines were projected onto the Schaefer atlas with 300 parcels,^[^ ^43^ ^]^ and the data were log‐transformed to create a structural connectivity matrix.

### Data Preprocessing—ABIDE

The imaging data were preprocessed using micapipe.^[^ ^101^ ^]^ The preprocessing steps were largely similar to those of the HCP minimal preprocessing pipeline. In summary, T1‐weighted MRI underwent gradient nonuniformity correction, non‐brain tissue removal, intensity normalization, and tissue segmentation. White and pial surfaces were generated, and inflation, topology correction, and spherical registration to the Conte69 template were performed.

The rs‐fMRI data were preprocessed as follows: the first five volumes were discarded, and head movements were corrected. Susceptibility distortion correction was conducted using main‐ and reverse‐phase encoded data. The FIX was then applied to eliminate nuisance variables.^[^ ^102^, ^103^, ^104^ ^]^ Volumetric time series data were mapped to the cortical surface, while spatial smoothing with a full width at half maximum of 10 mm was applied.

### Time Delay Matrix

Following the protocols of existing studies investigating latency structure,^[^ ^6^, ^18^ ^]^ a time delay matrix with a size of 314 × 314 was constructed from the preprocessed rs‐fMRI data for each participant. These regions consisted of cortical areas from the Schaefer atlas with 300 parcels^[^ ^43^ ^]^ and 14 subcortical regions from the Desikan–Killiany atlas.^[^ ^44^ ^]^ The lagged cross‐covariance was computed between a pair of time series data *x_i_* (*t*) and *x_j_* (*t*), where *i* and *j* denote the indices of the brain regions (Equation 1).

(1) $$C_{x_{i}x_{j}}\left(\tau \right)=\frac{1}{T}\int x_{i}\left(t+\tau \right)\cdot x_{j}\left(t\right)dt,\;\;i,j\in 1,2,\text{…},n$$

where *τ* represents the latency of time (i.e., time lag), *T* is the total time of the fMRI scan, and *n* is the number of brain regions. The *τ* that maximized $C_{x_{i}x_{j}}\left(\tau \right)$ was set as the latency *τ*_*i*,*j*_ between *i^th^* and *j^th^* regions. The time delay matrix is an antisymmetric *n* × *n* matrix, which had *τ*_*i*,*j*_ as its element that describes the covariance structure of the latency. Considering the hemodynamic response delays in fMRI signals, a 5 s threshold was applied to the latency value.^[^ ^6^, ^18^ ^]^ To assess the reliability of the latency eigenvectors across different temporal windows, latency eigenvectors were additionally generated with 3 and 1.5 s thresholds. In the ABIDE dataset, the time series were up‐sampled to 0.75 s (PITT) or 0.66 s (other sites) to match the TR of HCP (0.72 s) while constructing the time delay matrix.^[^ ^19^ ^]^

### Latency Structure Modeling

For each participant, low‐dimensional representations of the time delay matrix were generated using PCA and denoted the major components of the latency structure as latency eigenvectors. The primary goal of the dimensionality reduction algorithm was to represent the original data with fewer features, while retaining sufficient information. This approach helps to prevent multicollinearity and overfitting among features and reduces the complexity of the model. Group‐level template eigenvectors were defined based on a group‐averaged time delay matrix, while the eigenvectors of each participant were aligned to the template using Procrustes alignment.^[^ ^68^, ^105^ ^]^ The described approach was applied to the HCP data, with adjustments to suit the characteristics of the ABIDE dataset, as follows: the eigenvectors of each participant were aligned with the template eigenvectors derived from the group‐averaged time delay matrix of the ABIDE dataset, which were further matched to the HCP template eigenvectors.^[^ ^106^ ^]^ Age, sex, and site were controlled from the eigenvectors.^[^ ^86^, ^107^, ^108^ ^]^ To assess the robustness of the latency eigenvectors across different spatial granularities, additional latency eigenvectors were generated using the Schaefer atlas with 200 and 400 parcels and the Glasser multimodal atlas with 360 parcels.^[^ ^94^ ^]^ The robustness of the latency eigenvectors was also assessed using a different dimensionality reduction technique by generating latency eigenvectors using diffusion map embedding.^[^ ^50^ ^]^

### Neural Function Estimation Using a Biophysical Model

The pFIC model was employed to estimate neural‐level parameters. The conventional FIC model was extended by introducing spatial heterogeneity in synaptic parameters and noise amplitude to capture region‐specific excitatory‐inhibitory dynamics.^[^ ^24^ ^]^ The model describes neuronal activity using nonlinear differential equations that estimate excitatory (*E*) and inhibitory (*I*) neuronal populations as follows:

(2) $$I_{E,j}=w_{E}I_{0}+W_{EE}J_{NMDA}S_{E,j}+GJ_{NMDA}\sum_{k}C_{jk}S_{E,k}-W_{IE}S_{I,j}$$

(3) $$I_{I,j}=w_{I}I_{0}+W_{EI}J_{NMDA}S_{E,j}-W_{II}S_{I,j}\;$$

(4) $$r_{E,j}=\phi \left(I_{E,j}\right)=\frac{a_{E}I_{E,j}-b_{E}}{1-\exp\left(-d_{E}\left(a_{E}I_{E,j}-b_{E}\right)\right)}\;$$

(5) $$r_{I,j}=\phi \left(I_{I,j}\right)=\frac{a_{I}I_{I,j}-b_{I}}{1-\exp\left(-d_{I}\left(a_{I}I_{I,j}-b_{I}\right)\right)}\;$$

(6) $$\frac{dS_{E,j}}{dt}=-\frac{S_{E,j}}{\tau_{E}}+\left(1-S_{E,j}\right)\gamma r_{E,j}+\sigma \nu_{j}\left(t\right)$$

(7) $$\frac{dS_{I,j}}{dt}=-\frac{S_{I,j}}{\tau_{I}}+r_{I,j}+\sigma \nu_{j}\left(t\right)$$

where *I* is synaptic currents, *r* is firing rate, and *S* is synaptic gating variable. Equations (2) and (3) optimize synaptic currents using fixed parameters. The input current *I*_*E*,*j*_ of the excitatory population of the *j^th^* brain region is the sum of external input current *w_E_I*_0_ (*w*: input current strength, *I*_0_: baseline input current), intra‐regional excitatory‐to‐excitatory recurrent connection strength *W_EE_* scaled by the synaptic coupling constant *J_NMDA_*, inter‐regional input controlled by the structural connectivity between *j^th^* and *k^th^* brain regions (*C_jk_*) scaled by a global scaling factor *G*, and intra‐regional negative feedback governed by the inhibitory‐to‐excitatory connection strength *W_IE_*.^[^ ^24^, ^27^ ^]^ The input current $I_{I,j}$ of the inhibitory population is determined by the input current, excitatory‐to‐inhibitory connection strength, and inhibitory‐to‐inhibitory connection strength.^[^ ^24^, ^27^ ^]^ The input currents ($I_{E,j}$, $I_{I,j}$) were fed into the sigmoid function in Equations (4) and (5) to determine the firing rate of the neuronal population. The parameters *a*,  *b*,  *d* were fixed as in the previous study.^[^ ^27^ ^]^ Finally, Equations (6) and (7) compute the synaptic gating variables (*S*_*E*,*j*_,  *S*_*I*,*j*_), with the fixed kinetic parameters for synaptic activities (*τ*_*E*_,*τ*_*I*_, and *γ*) as defined in the previous study.^[^ ^27^ ^]^ The variables *v_j_* (*t*) and σ denote uncorrelated standard Gaussian noise and its corresponding amplitude.

For each brain region, the E/I ratio was defined as the ratio of the temporal average of excitatory and inhibitory synaptic gating variables (*S_E_* and *S_I_*).^[^ ^24^ ^]^ The excitatory synaptic gating variable (*S_E_*) was fed into the Balloon–Windkessel hemodynamic model to generate BOLD signals.^[^ ^27^, ^109^, ^110^ ^]^ The pFIC model optimized excitatory‐to‐excitatory recurrent connection weight (*W_EE_*) and excitatory‐to‐inhibitory connection weight (*W_EI_*) using a linear combination of T1w/T2w intensity ratio‐based myelin map and the first functional gradient (see *Functional gradients* section below). The parameters of *j* ^th^ brain region were defined using the following equations:

(8) $$W_{EE,j}=a+b\times \mathrm{myeli}n_{j}+c\times \mathrm{functional}\;\mathrm{gradien}t_{j}$$

(9) $$W_{EI,j}=d+e\times \mathrm{myeli}n_{j}+f\times \mathrm{functional}\;\;\mathrm{gradien}t_{j}$$

(10) $$\sigma_{j}=g+h\times \mathrm{myeli}n_{j}+i\times \mathrm{functional}\;\;\mathrm{gradien}t_{j}$$

The estimated neural parameters, including excitatory synaptic gating (*S_E_*), inhibitory synaptic gating (*S_I_*), excitatory‐to‐excitatory recurrent connection strength (*W_EE_*), excitatory‐to‐inhibitory recurrent connection strength (*W_EI_*), and E/I ratio, were associated with latency eigenvectors using 1000 spin permutation tests.^[^ ^45^ ^]^ The multiple comparisons were corrected using an FDR.^[^ ^46^ ^]^ Furthermore, to assess the reliability of the fMRI‐derived latency eigenvectors, their spatial patterns were compared with those calculated from BOLD signals with faster TRs (0.36, 0.18, and 0.09 s) by calculating spatial correlations.

### Intrinsic Neural Timescale

The INT for each region was estimated from the rs‐fMRI data. The INT is the period during which neural activity within a specific brain region correlates with itself. Following a previous study, a nonlinear exponential decay function with an offset was fitted to the empirically estimated autocorrelation function *R*(*k*Δ), ^[^ ^28^ ^]^ defined as:

(11) $$R\left(k\Delta \right)=A\left[\exp\left(-\frac{k\Delta }{T}\right)+B\right]$$

where *k*Δ represents the time bins (i.e., *k*Δ = |timepoint index *i* − timepoint index *j*|), *A* represents the scaling factor, *B* is the offset for the contribution of timescales much longer than the observation window, and *T* denotes the INT (i.e., the rate of autocorrelation decay). The hemodynamic response delays of fMRI were considered, and a 5 s threshold was applied.^[^ ^6^, ^18^ ^]^ To identify the link between the latency structure and INT, these two features were associated using 1000 spin permutation tests,^[^ ^45^ ^]^ while multiple comparisons were corrected using an FDR.^[^ ^46^ ^]^ INT was also predicted using latency eigenvectors to enhance the identification.

### Functional Gradients

The cortical hierarchy of latency structure was further investigated using functional gradient analysis. Without considering latency, Pearson's correlation coefficients were calculated between the time series of different brain regions and Fisher's r‐to‐z transformed.^[^ ^111^ ^]^ Low‐dimensional representations of functional connectivity (i.e., gradients) were generated using established dimensionality reduction techniques implemented in the BrainSpace toolbox (https://github.com/MICA‐MNI/BrainSpace).^[^ ^50^, ^68^ ^]^ Specifically, a cosine similarity kernel was employed to compute an affinity matrix to capture the interregional similarity of functional connectivity. Only the top 10% of elements in each row were retained, and diffusion map embedding, a nonlinear dimensionality reduction technique was applied.^[^ ^50^ ^]^ The individual functional gradients were aligned to the group‐level template gradients, defined based on a group‐averaged functional connectivity matrix using Procrustes alignment.^[^ ^68^, ^105^ ^]^ Each latency eigenvector was further correlated with functional gradients using 1000 spin permutations, and multiple comparisons were corrected using FDR.^[^ ^46^ ^]^

### Alterations in the Latency Eigenvectors in Autism Spectrum Disorder

The first few latency eigenvectors were compared between the ASD and TD groups using Hotelling's T^2^ test implemented in the BrainStat toolbox (https://github.com/MICA‐MNI/BrainStat).^[^ ^112^ ^]^ A total of 1000 permutation tests were conducted by randomly assigning each subject to each group, and multiple comparisons were corrected using an FDR.^[^ ^45^, ^46^ ^]^ The between‐group differences were stratified according to seven intrinsic functional networks, including visual, somatomotor, dorsal attention, ventral attention, limbic, frontoparietal, and default mode networks.^[^ ^8^ ^]^ Meta‐analysis‐based cognitive decoding was further performed, whose cognitive terms were related to the spatial maps of the between‐group differences.^[^ ^48^, ^49^ ^]^

### Statistical Analysis

Comparisons of latency eigenvectors with neural parameters derived from the biophysical model, INT, and functional gradients were conducted by calculating Pearson's correlations. Statistical significance was determined based on 1000 spin permutation tests,^[^ ^45^ ^]^ and multiple comparisons were corrected using an FDR.^[^ ^46^ ^]^ Between‐group differences in latency eigenvectors between individuals with ASD and TD were assessed using Hotelling's T^2^ test. The significance of the group difference was evaluated using 1000 permutation tests, where subjects were randomly assigned to either the ASD or TD groups, and an FDR was applied to correct for multiple comparisons. The prediction of INT using latency eigenvectors was performed using ordinary least squares linear regression.

## Conflict of Interest

The authors declare no conflict of interest.

## Code Availability Statement

The codes for the latency structure are available at https://github.com/gudtls17/LatencyStructure (Python version) and https://gitlab.com/by9433/lagstructure (MATLAB version), the codes for the pFIC model at https://github.com/ThomasYeoLab/CBIG, the codes for INT calculation at https://github.com/gudtls17/LatencyStructure, the codes for gradient generation at https://github.com/MICA‐MNI/BrainSpace, and the codes for statistical analyses are available at https://github.com/MICA‐MNI/BrainStat.

## Supporting information



Supporting Information

## Data Availability

Imaging and phenotypic data were provided in part by the Human Connectome Project (HCP; https://www.humanconnectome.org). Imaging and phenotypic data were provided, in part, by the Autism Brain Imaging Data Exchange initiative (ABIDE; https://www.fcon‐1000.projects.nitrc.org/indi/abide/).
