# Supplementary material for: Spatiotemporal Characterization of the Functional MRI Latency Structure with Respect to Neural Signaling and Brain Hierarchy
Source: Adv Sci (Weinh). 2025 Aug 27;12(43):e04956. doi: 10.1002/advs.202504956 (PMC12631850; doi:10.1002/advs.202504956)
Supplement: Supplementary file 1 — Supporting Information [file ADVS-12-e04956-s001.docx]

**Supplementary Information**

**Supplementary Table 1. MRI acquisition parameters.**

| **Categories** | **HCP** | | | **CALTECH** | | | | **CMU** | | | | **NYU** | | | | **PITT** | | |
| --- | --- | --- | --- | --- | --- | --- | --- | --- | --- | --- | --- | --- | --- | --- | --- | --- | --- | --- |
| Scanner | Siemens 3T Skyra | | Siemens magnetom TrioTim syngo MR B17 | | | | Siemens magnetom Verio syngo MR B17 | | | | Siemens magnetom  Allegra syngo MR 2004A | | | | Siemens magnetom  Allegra syngo MR A30 | | | |
| Modality | T1w | rs-fMRI | T1w | | rs-fMRI | | T1w | | rs-fMRI | | T1w | | rs-fMRI | | T1w | | rs-fMRI | |
| TR (ms) | 2400 | 720 | 1590 | | 2000 | | 1870 | | 2000 | | 2530 | | 2000 | | 2100 | | 1500 | |
| TE (ms) | 2.14 | 33.1 | 2.73 | | 30 | | 2.48 | | 30 | | 3.25 | | 15 | | 3.93 | | 25 | |
| Slices | 256 | 72 | 176 | | 34 | | 176 | | 28 | | 128 | | 33 | | 176 | | 29 | |
| Flip angle (°) | 8 | 52 | 10 | | 75 | | 8 | | 73 | | 7 | | 90 | | 7 | | 70 | |
| FoV (mm^2^) | 224 × 224 | 208 × 180 | 256 × 256 | | 224 × 224 | | 256 × 256 | | 192 × 192 | | 256 × 256 | | 240 × 192 | | 269 × 269 | | 200 × 200 | |
| Resolution (mm^3^) | 0.7×0.7×0.7 | 2.0×2.0×2.0 | 1.0×1.0×1.0 | | 3.5×3.5×3.5 | | 1.0×1.0×1.0 | | 3.0×3.0×3.0 | | 1.3×1.0×1.3 | | 3.0×3.0×4.0 | | 1.1×1.1×1.1 | | 3.1×3.1×4.0 | |
| **Categories** |  | | | **TRINITY** | | | | **UM_2** | | | | **USM** | | | | **YALE** | | |
| Scanner |  | | | Philips 3T Achieva | | | | 3T GE Signa | | | | Siemens magnetom  TrioTim syngo MR B17 | | | | Siemens magnetom  TrioTim syngo MR B17 | | |
| Modality | - | | | T1w | | rs-fMRI | | T1w | | rs-fMRI | | T1w | | rs-fMRI | | T1w | | rs-fMRI |
| TR (ms) | - | | | 8.5 | | 2000 | | - | | 2000 | | 2300 | | 2000 | | 1230 | | 2000 |
| TE (ms) | - | | | 3.9 | | 28 | | 1.8 | | 30 | | 2.91 | | 28 | | 1.73 | | 25 |
| Slices | - | | | 160 | | 39 | | 128 | | 40 | | 160 | | 40 | | 176 | | 34 |
| Flip angle (°) | - | | | 8 | | 90 | | 15 | | 90 | | 9 | | 90 | | 9 | | 60 |
| FoV (mm^2^) | - | | | 256 × 256 | | 240 × 240 | | 260 × 208 | | 220 × 220 | | 256 × 240 | | 220 × 220 | | 250 × 250 | | 220 × 220 |
| Resolution (mm^3^) | - | | | 1.0×1.0×1.0 | | 3.0×3.0×3.5 | | - | | 3.438×3.438×3.0 | | 1.0×1.0×1.2 | | 3.4×3.4×3.0 | | 1.0×1.0×1.0 | | 3.4×3.4×4.0 |

*Abbreviations:* MRI, magnetic resonance imaging; TR, repetition time; TE, time to echo; T1w, T1-weighted; rs-fMRI, resting-state functional magnetic resonance imaging; FoV, field of view;


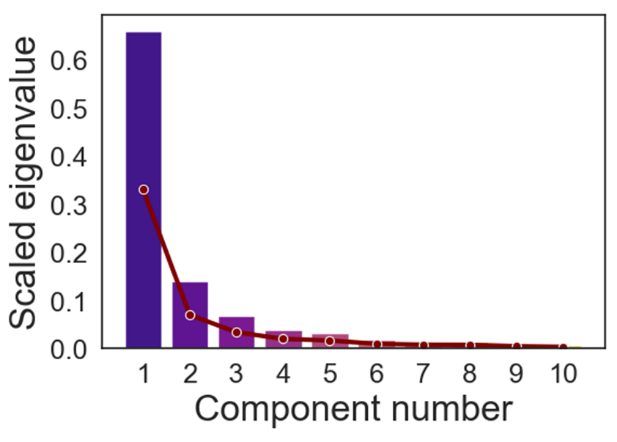


**Supplementary Figure 1. Scaled explained variance with varying numbers of eigenvectors.
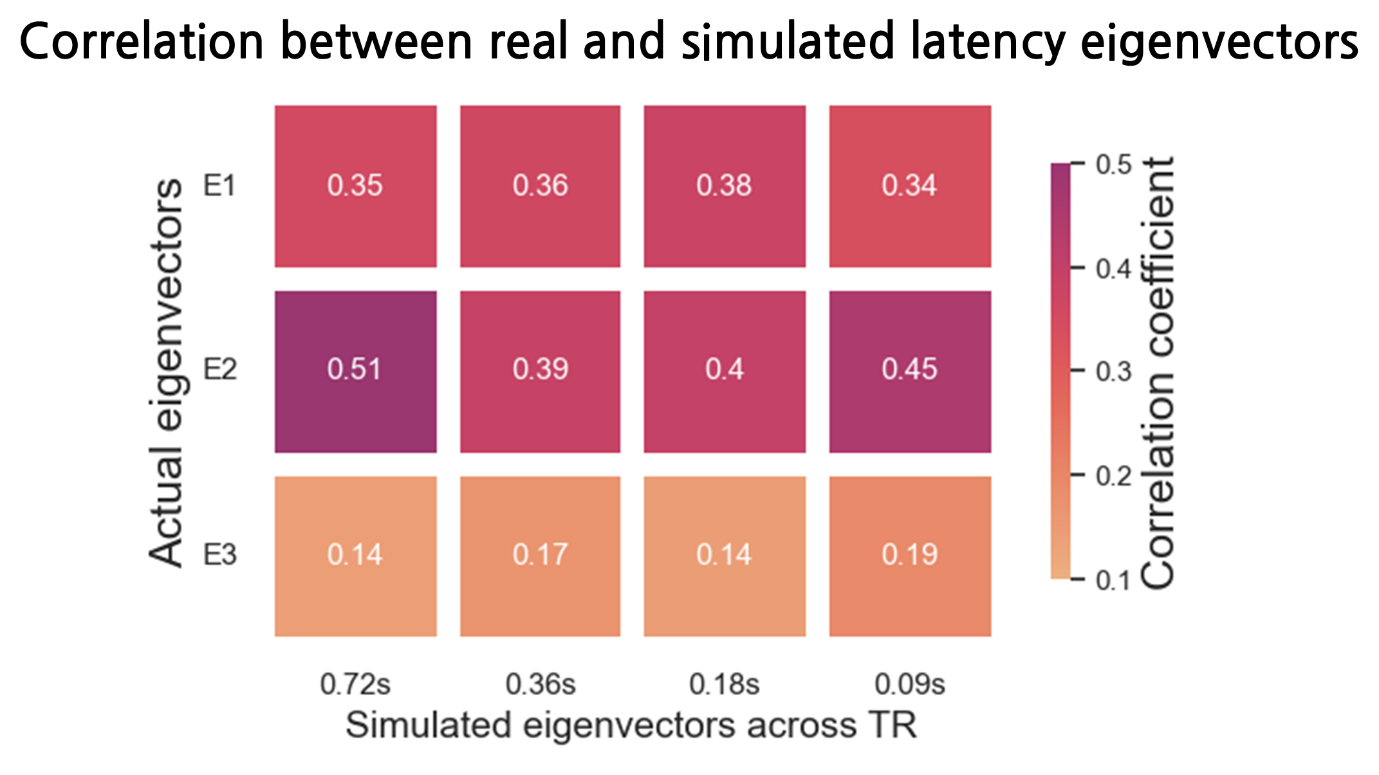
**

**Supplementary Figure 2. Comparison of the spatial patterns of actual and simulated eigenvectors across different repetition times (TRs).** Correlations between pairs of latency eigenvectors are calculated by comparing actual and simulated data with varying TRs.


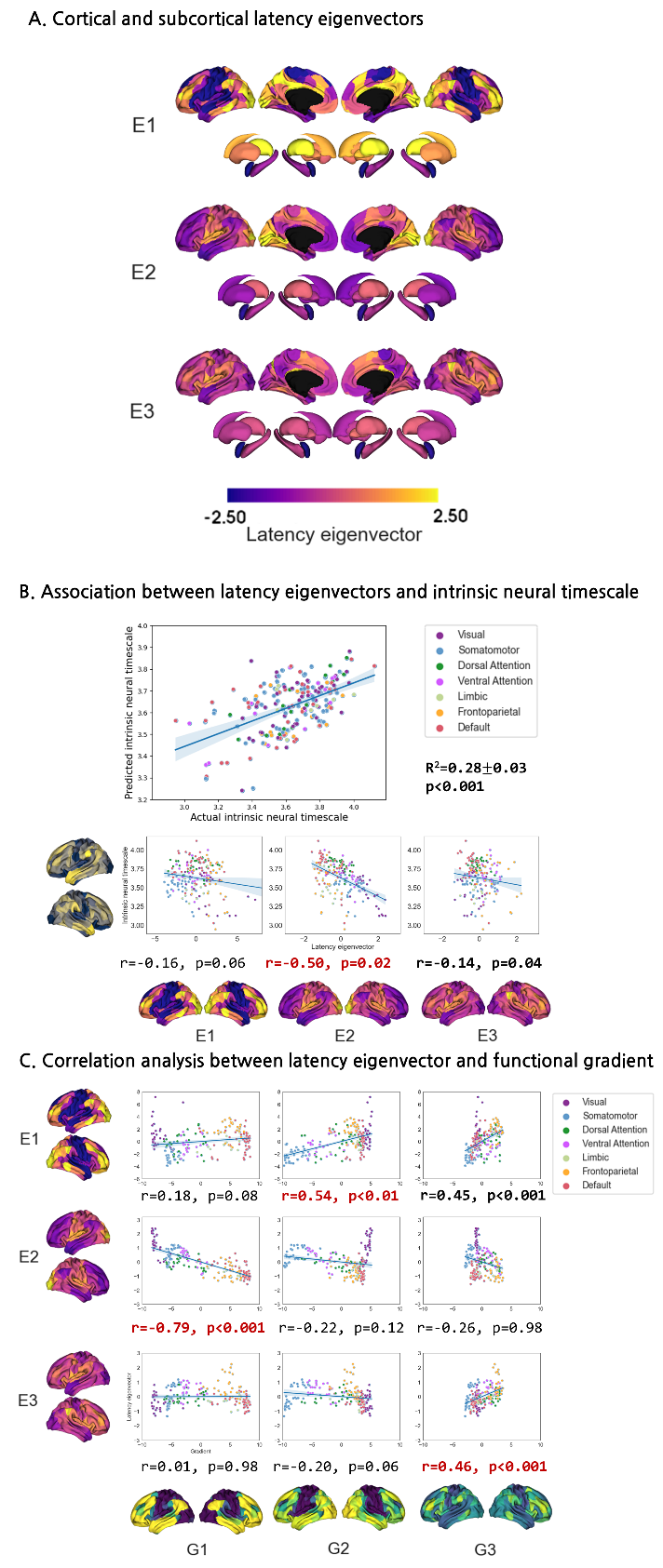


**Supplementary Figure 3. Latency eigenvectors and association results mapped using the Schaefer atlas with 200 parcels.** For details, see *Figures 1–3*.


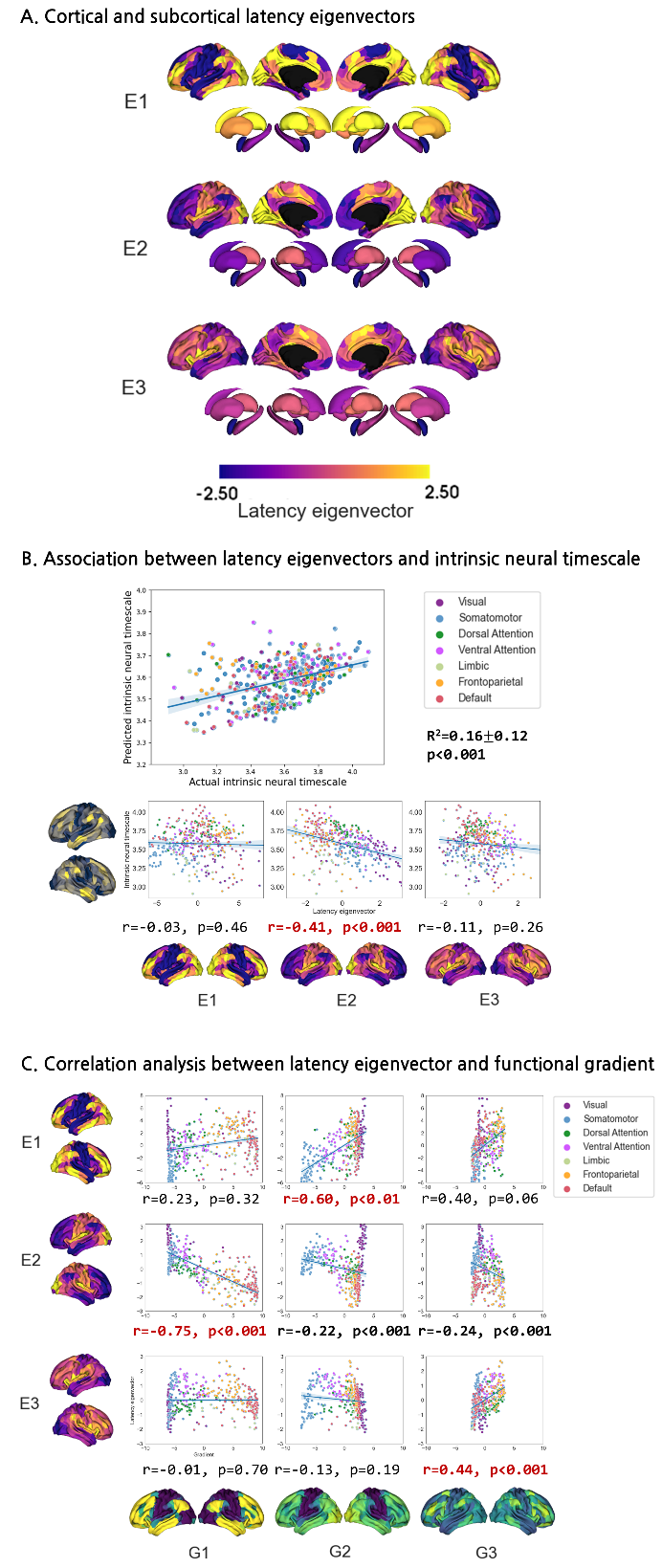


**Supplementary Figure 4. Latency eigenvectors and association results mapped using the Schaefer atlas with 400 parcels.** For details, see *Figures 1–3*.


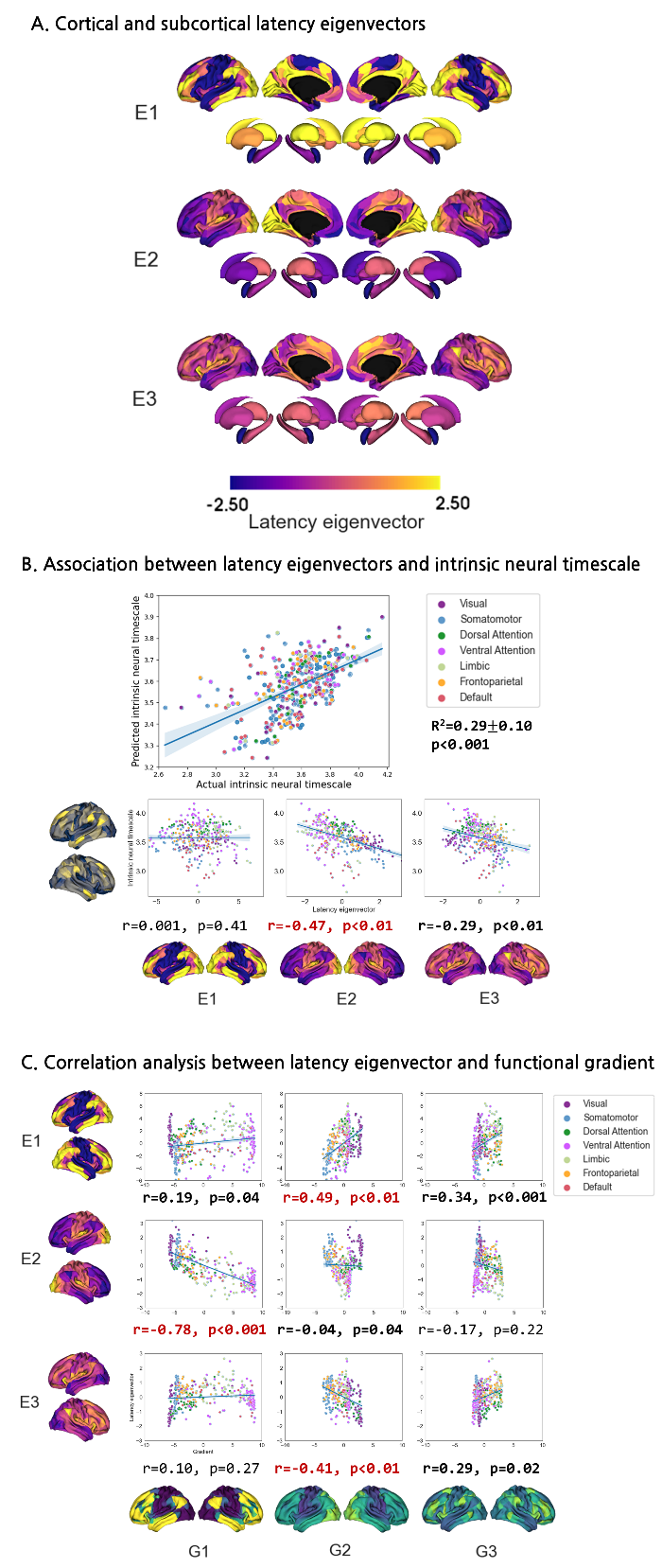


Supplementary Figure 5. Latency eigenvectors and association results mapped using the Glasser atlas with 360 parcels. **For details, see *Figures 1–3*.**


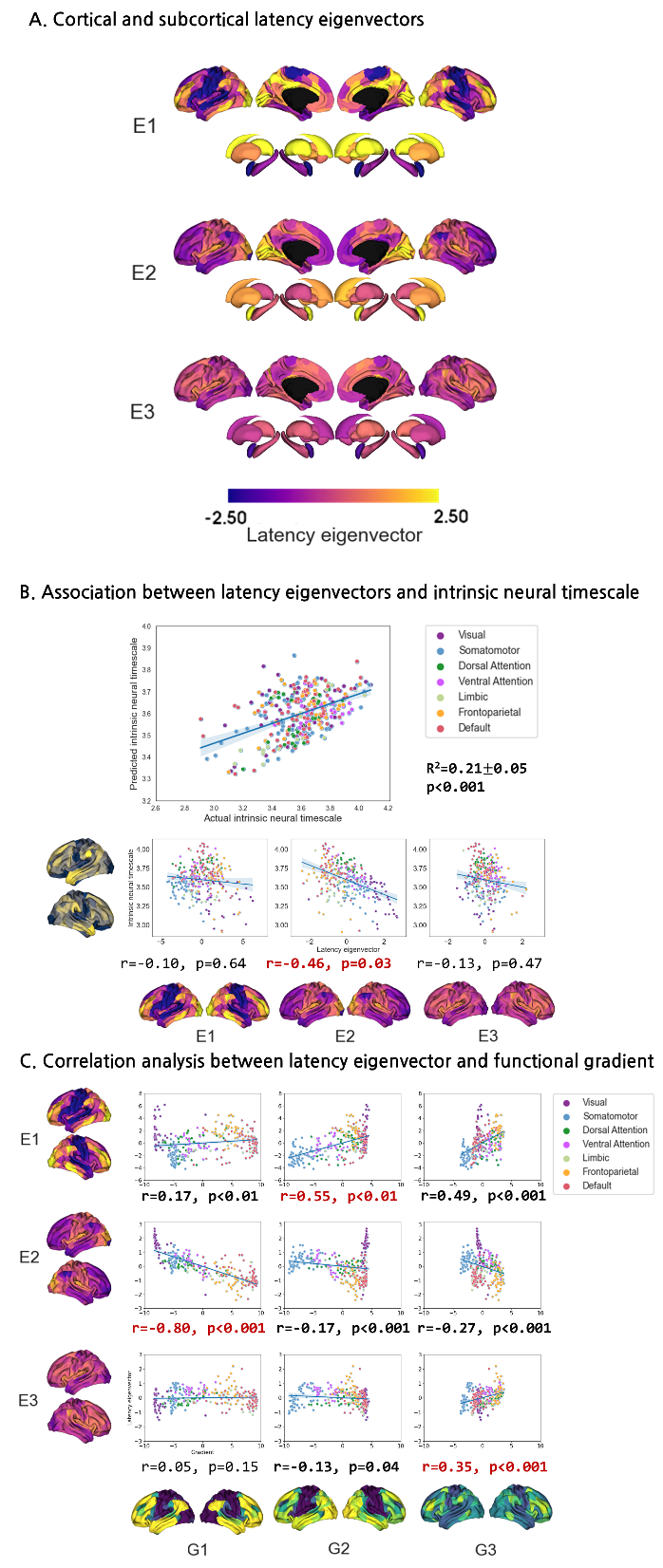


**Supplementary Figure 6. Latency eigenvectors and association results derived using the 3-s threshold of the time delay matrix.** For details, see *Figures 1–3*.

**
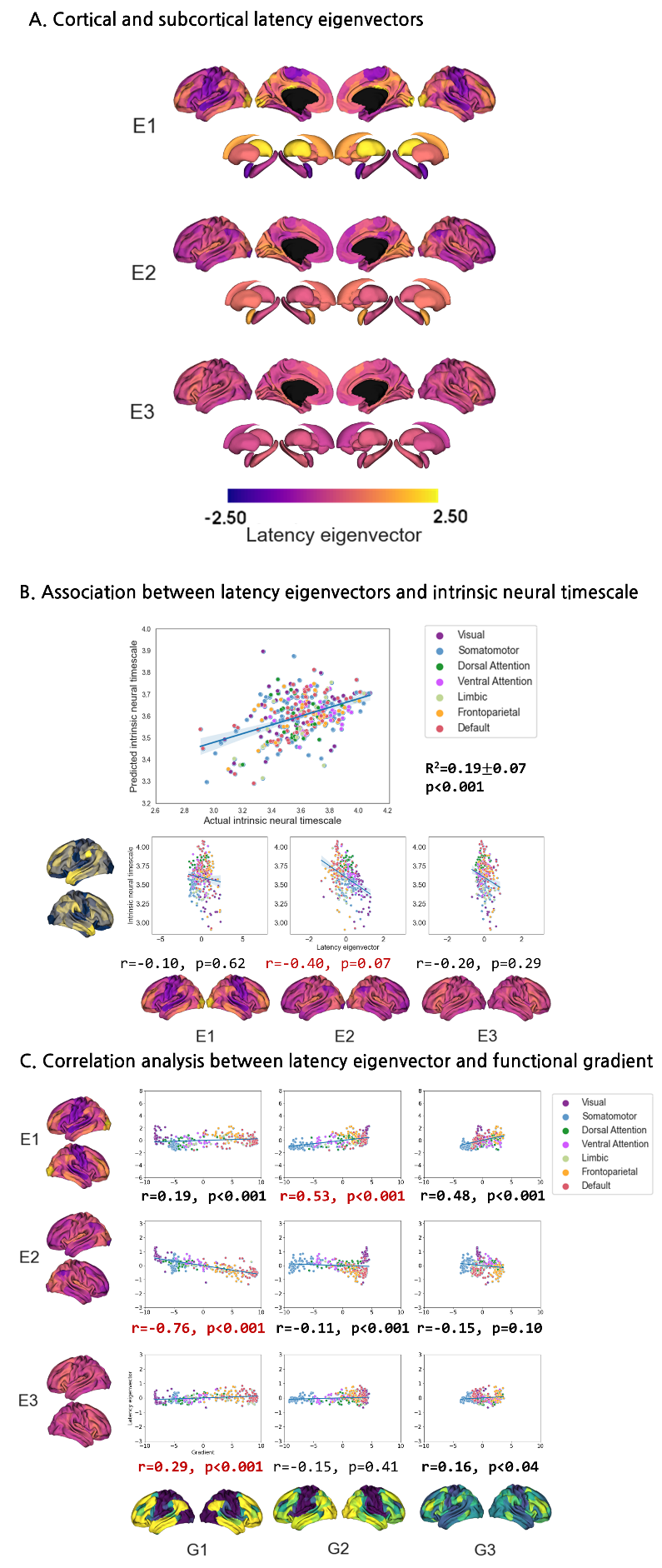
**

**Supplementary Figure 7. Latency eigenvectors and association results derived using the 1.5-s threshold of the time delay matrix.** For details, see *Figures 1–3*.

**
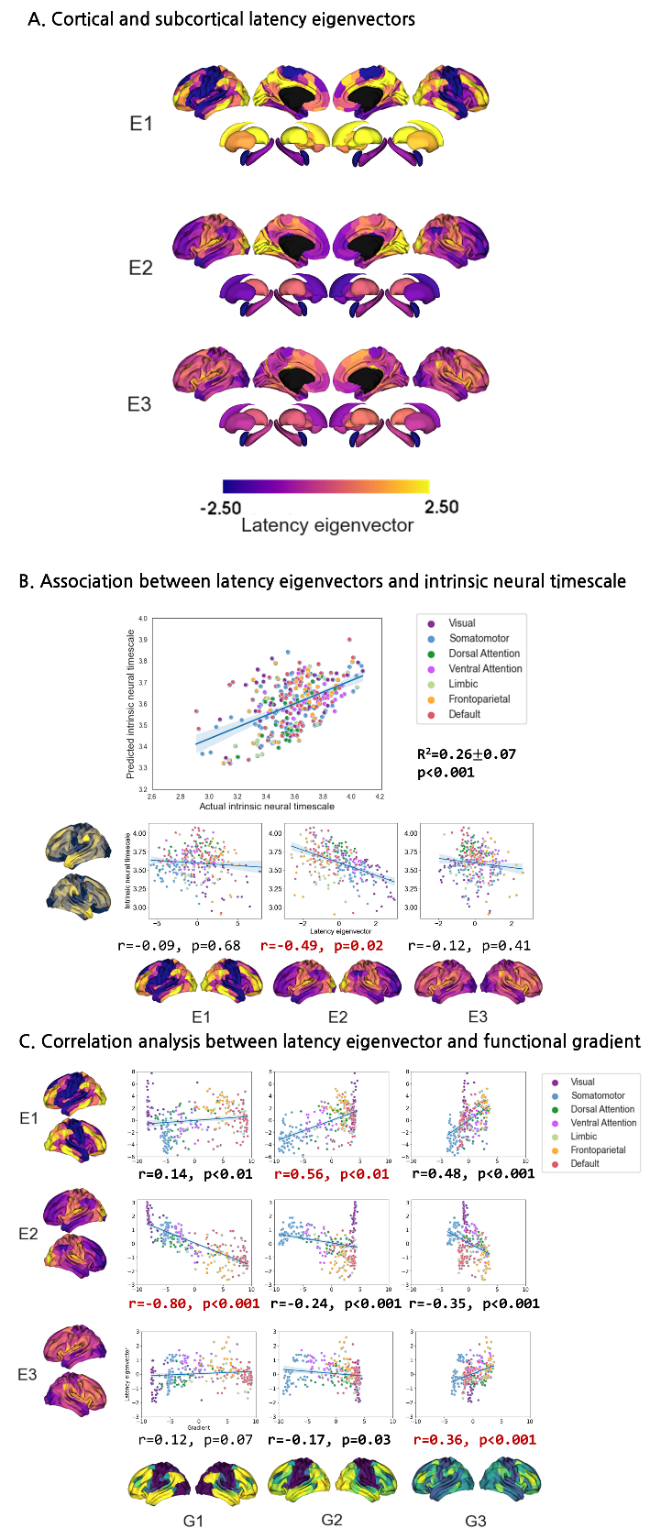
**

**Supplementary Figure 8. Latency eigenvectors and association results derived using the non-linear dimensionality reduction method (i.e., diffusion map embedding).** For details, see *Figures 1–3*.
